# Acinar Cell Carcinoma of the Pancreas: Overview of Clinicopathologic Features and Insights into the Molecular Pathology

**DOI:** 10.3389/fmed.2015.00041

**Published:** 2015-06-15

**Authors:** Stefano La Rosa, Fausto Sessa, Carlo Capella

**Affiliations:** ^1^Department of Pathology, Ospedale di Circolo, Varese, Italy; ^2^Department of Surgical and Morphological Sciences, University of Insubria, Varese, Italy

**Keywords:** pancreas, acinar cell carcinoma, morphology, immunohistochemistry, molecular pathology

## Abstract

Acinar cell carcinomas (ACCs) of the pancreas are rare pancreatic neoplasms accounting for about 1–2% of pancreatic tumors in adults and about 15% in pediatric subjects. They show different clinical symptoms at presentation, different morphological features, different outcomes, and different molecular alterations. This heterogeneous clinicopathological spectrum may give rise to difficulties in the clinical and pathological diagnosis with consequential therapeutic and prognostic implications. The molecular mechanisms involved in the onset and progression of ACCs are still not completely understood, although in recent years, several attempts have been made to clarify the molecular mechanisms involved in ACC biology. In this paper, we will review the main clinicopathological and molecular features of pancreatic ACCs of both adult and pediatric subjects to give the reader a comprehensive overview of this rare tumor type.

## Background

Acinar cell carcinomas (ACCs) of the pancreas are very rare pancreatic neoplasms accounting for approximately 1–2% of pancreatic tumors in adults and about 15% in pediatric subjects (1). Although they are traditionally considered as a single tumor entity, several recent findings have demonstrated that they may show different clinical symptoms at presentation, different morphological features, different outcomes, and different molecular alterations. This heterogeneous clinicopathological spectrum may give rise to difficulties in the clinical and pathological diagnosis with consequential therapeutic and prognostic implications. The differential diagnosis from ductal adenocarcinomas is generally easy and is mainly based on morphology, whereas the distinction from pancreatic neuroendocrine tumors (PanNETs) is more difficult because of some similar morphological features. In addition, the differential diagnosis from solid pseudopapillary neoplasms and pancreatoblastomas may be problematic. The diagnostic hallmark of ACC is the immunohistochemical demonstration of acinar-specific products such as trypsin, lipase, amylase, and carboxyl ester lipase (CEL). The molecular mechanisms involved in ACC pathogenesis and progression are largely unknown, although in recent years, several attempts have been made to better understand the molecular pathology of such rare cancers. In general, the typical abnormalities of ductal adenocarcinomas including mutations in *KRAS*, *DPC4*, *p16*, and *TP53* genes are absent or very rare in ACCs, while the better known pathogenetic mechanism includes abnormalities in the APC/β-catenin pathway (1).

In this paper, we will review the main clinicopathological and molecular features of pancreatic ACCs to give the reader a comprehensive overview of this rare tumor type.

## Acinar Cell Carcinoma in Adults

### Clinical features

The average age of adult patients is approximately 59 years old (range 20–88 years). Males are more commonly affected with a male/female ratio of 2:1 (2–4). In the majority of cases presenting symptoms are non-specific and include abdominal pain, weight loss, vomiting, and nausea, which are related to tumor growth and/or metastatic spread. Jaundice can be present, but it is much rarer than in ductal adenocarcinoma (2, 4). Patients with metastatic disease rarely show symptoms due to lipase hypersecretion, which include subcutaneous fat necrosis and polyarthralgia (5–7). Occasionally, patients, especially when young, may present increased alpha-fetoprotein (AFP) blood levels that should be considered as a suspicious marker of ACC in the presence of a pancreatic mass (8–11). Although most ACCs arise sporadically, rare cases diagnosed in the context of Lynch syndrome or familial adenomatous polyposis (FAP) have been documented (4, 12–14).

### Macroscopy

**Figure 1:**
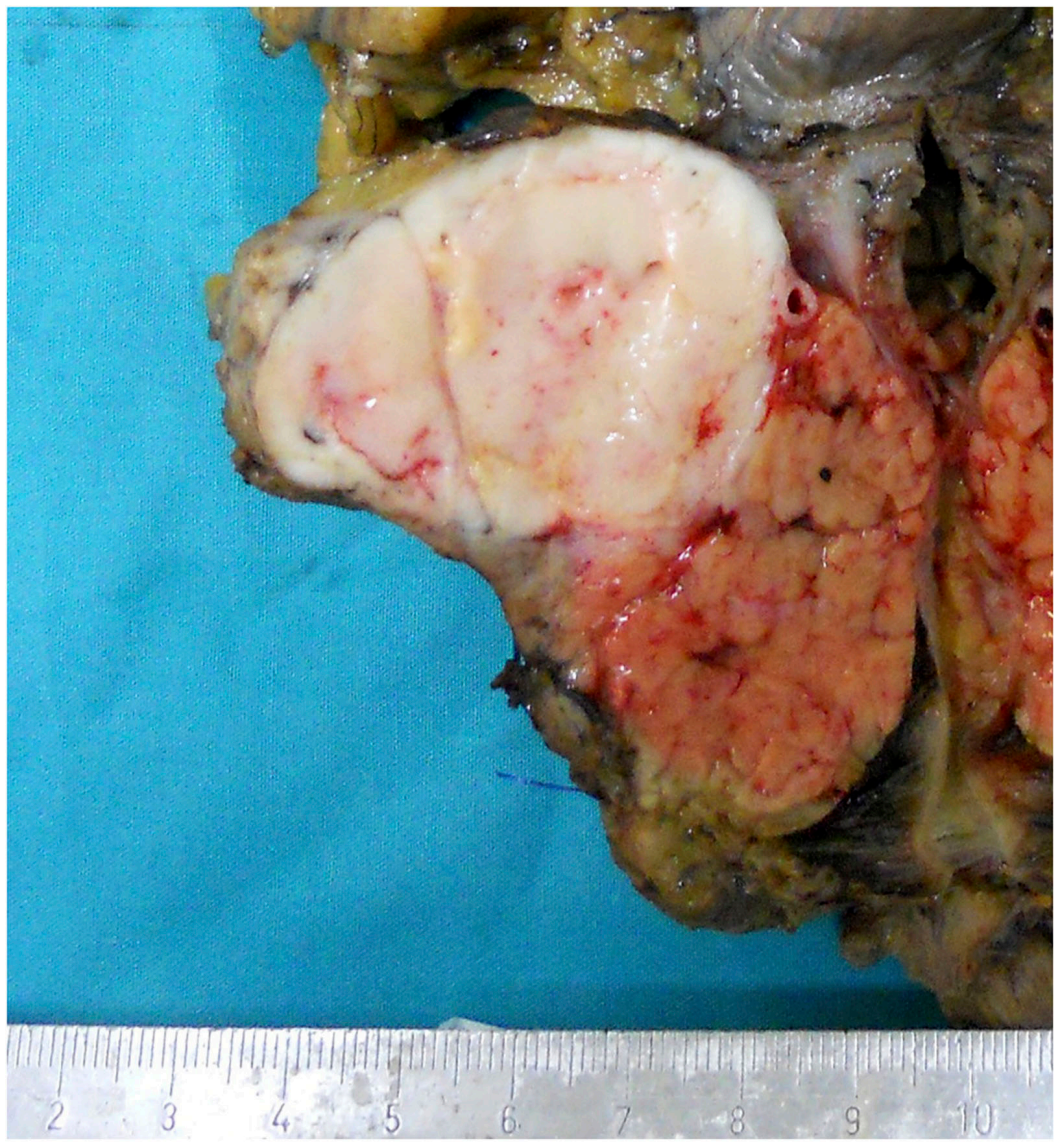
**Macroscopic appearance of a well circumscribed and large acinar cell carcinoma of the pancreatic head**.

Acinar cell carcinomas may arise in any portion of the pancreas. In a recently reported series in which macroscopic information was available for 58 ACCs, 22 tumors were in the head, 2 interested the head and the body, 5 the body, 12 the body and tail, 16 the tail, and 1 the entire pancreas (4). Tumors are generally large (average diameter of 8–10 cm), well circumscribed, and at least partially encapsulated. The cut surface generally appears homogeneous pink to tan, fleshy, or even friable in consistency (Figure 1). Hemorrhage and necrosis can be observed as well as cystic changes. A rare variant of ACC, exclusively characterized by variable-sized cysts, has been named acinar cell cystadenocarcinoma (15). Invasion of the tumor through the capsule is a common finding and in about 50% of cases infiltration of the duodenum, large vessels, stomach, kidney, peritoneum, or spleen can also be observed (1, 4).

### Histological features

**Figure 2:**
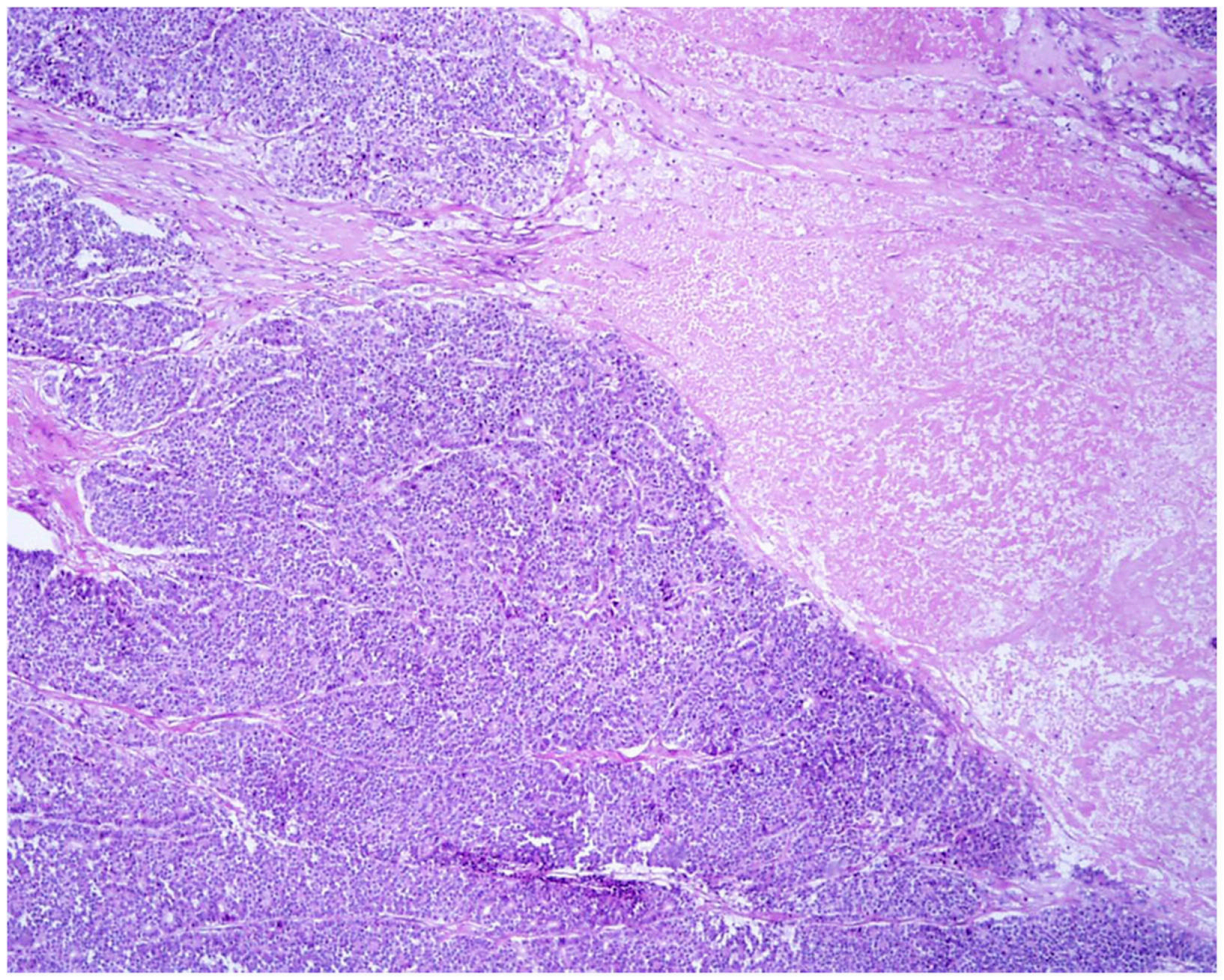
**At scan magnification, most acinar cell carcinomas appear highly cellular with a lobular architecture and scant fibrous stroma**. Abundant necrosis (right) is frequently observed. These morphological features can help in the differential diagnosis with the more common ductal adenocarcinoma that is generally less cellular, shows abundant fibrous stroma without or with focal necrosis.

**Figure 3:**
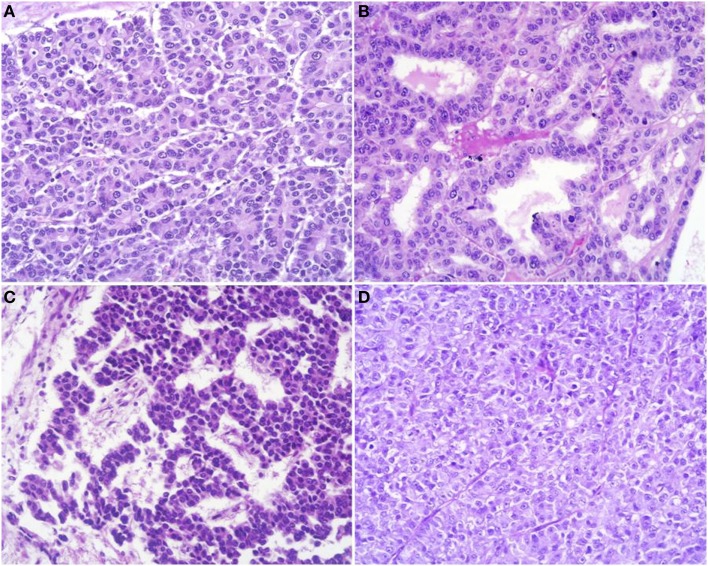
**Acinar cell carcinomas may have different histological features**. The acinar architectural pattern **(A)** is characterized by cells forming structures resembling normal pancreatic acini, sometimes with minute lumens. Cells are in a monolayer with basally located nuclei and have a moderate amount of granular eosinophilic cytoplasm. The glandular pattern **(B)** is characterized by proliferation of cells, sometimes arranged in multiple layers, forming glandular structures. The trabecular pattern **(C)** is characterized by trabecular structures formed by ribbons of cells strongly resembling the morphology of pancreatic neuroendocrine tumors. The solid pattern **(D)** is characterized by large sheets of poorly differentiated cells without lumens that generally show large nuclei with dispersed chromatin and prominent nucleoli.

**Figure 4:**
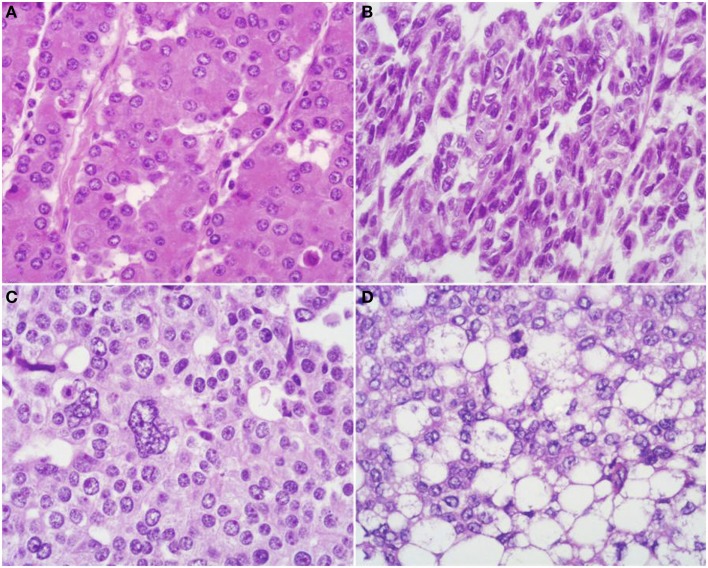
**Uncommon histological features of pancreatic acinar cell carcinomas include oncocytic cells (A), spindle cells (B), pleomorphic cells (C), and clear cells (D)**.

At scan magnification, tumors appear highly cellular with a lobular architecture and scant fibrous stroma (Figure 2). Necrosis is frequent and in about 1/3 of cases is prominent. These morphological features can be useful for the differential diagnosis with the more common ductal adenocarcinomas that are generally less cellular, and show abundant fibrous stroma without or with focal necrosis (16). ACCs may have different histological features, ranging from acinar structures similar to normal pancreatic acini to solid growths composed of large sheets of poorly differentiated neoplastic cells. The acinar architectural pattern (Figure 3A) is characterized by cells forming structures resembling normal acini, sometimes with minute lumens. Cells are in a monolayer with basally located nuclei and have a moderate amount of granular eosinophilic cytoplasm. In some cases, the lumens can be dilated resembling glandular structures with cells sometimes arranged in multiple layers (glandular pattern, Figure 3B). The trabecular pattern (Figure 3C) is characterized by trabecular structures formed by ribbons of cells strongly resembling the morphology of PanNETs. The solid pattern (Figure 3D) is characterized by large sheets of cells without lumens that generally show large nuclei with dispersed chromatin and prominent nucleoli. With the exception of the typical acinar architecture, the other three patterns of growth may be difficult to interpret because they resemble those of other pancreatic neoplasms including solid pseudopapillary tumors (SPTs), PanNETs, and ductal adenocarcinomas. In these cases, immunohistochemistry is mandatory for the correct diagnosis (see the next paragraph). The most frequent morphologies are represented by acinar and solid growths, although a mixture of patterns can be frequently found within an individual ACC. Nuclei are generally uniform and a single nucleolus is characteristic. Mitotic rate is variable but generally high with a recently reported mean mitotic index of 14.63 (4). The cytoplasm is abundant, finely granular, and eosinophilic. It also contains several zymogen granules, which are periodic acid-Schiff (PAS) positive and resistant to diastase digestion (dPAS). In addition to these histological features, uncommon variants (Figure 4) have been described recently including oncocytic, spindle, and pleomorphic types (4). These rare variants should be taken into account because can give diagnostic difficulties, especially when looking at small bioptic specimens. They enter in the differential diagnosis with the more common oncocytic PanNETs (17) and undifferentiated carcinomas (18). Moreover, clear cell ACCs have been described anecdotally and recently observed by us in a liver metastasis and by Dr. Mounajjed (Mayo Clinic, Rochester, MN, USA) in a primary pancreatic ACC. These very uncommon ACC types enter in the differential diagnosis with clear cell PanNETs that can be observed sporadically or in association with both von Hippel–Lindau disease and MEN1 (19, 20). Intraductal dissemination of ACC, characterized by intraductal polypoid growth, has been described (21–23). Interestingly, it has been suggested that ACCs showing this peculiar pattern of growth may be associated with less aggressive clinicopathologic features (23).

#### Histological Variant of ACC

##### Mixed acinar-neuroendocrine carcinomas

**Figure 5:**
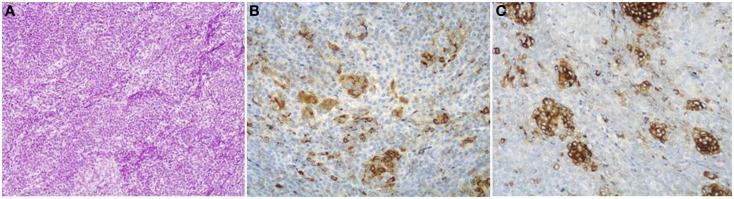
**Mixed acinar-neuroendocrine carcinomas (MANECs) are composed of both acinar and neuroendocrine components and each must represent at least 30% of the tumor tissue**. The neuroendocrine component is very difficult to identify morphologically on H&E stained sections **(A)**. The use of immunohistochemistry employing antibodies directed against general neuroendocrine [**(B)** chromogranin A] and acinar markers [**(C)** BCL10] is mandatory for the diagnosis.

About one-third of ACCs shows a significant neuroendocrine component (>30%) and these cases are defined as mixed acinar-neuroendocrine carcinomas (MANECs) (24). The neuroendocrine component is very difficult to identify morphologically on H&E stained sections and the use of immunohistochemistry employing antibodies directed against general neuroendocrine markers (chromogranin A and synaptophysin) is mandatory (Figure 5). However, it is worth noting that the survival rate of patients with ACCs and MANECs is the same (4).

##### Acinar cell cystadenocarcinoma

This rare variant presents as large, encapsulated multicystic lesion, measuring up to 25 cm in diameter. This neoplasm is composed of acinar and microglandular complexes as well as microcysts and macrocysts. The cysts are lined by cells with acinar morphology that are immunoreactive for pancreatic enzymes (15, 25, 26).

##### Mixed acinar-ductal adenocarcinoma

**Figure 6:**
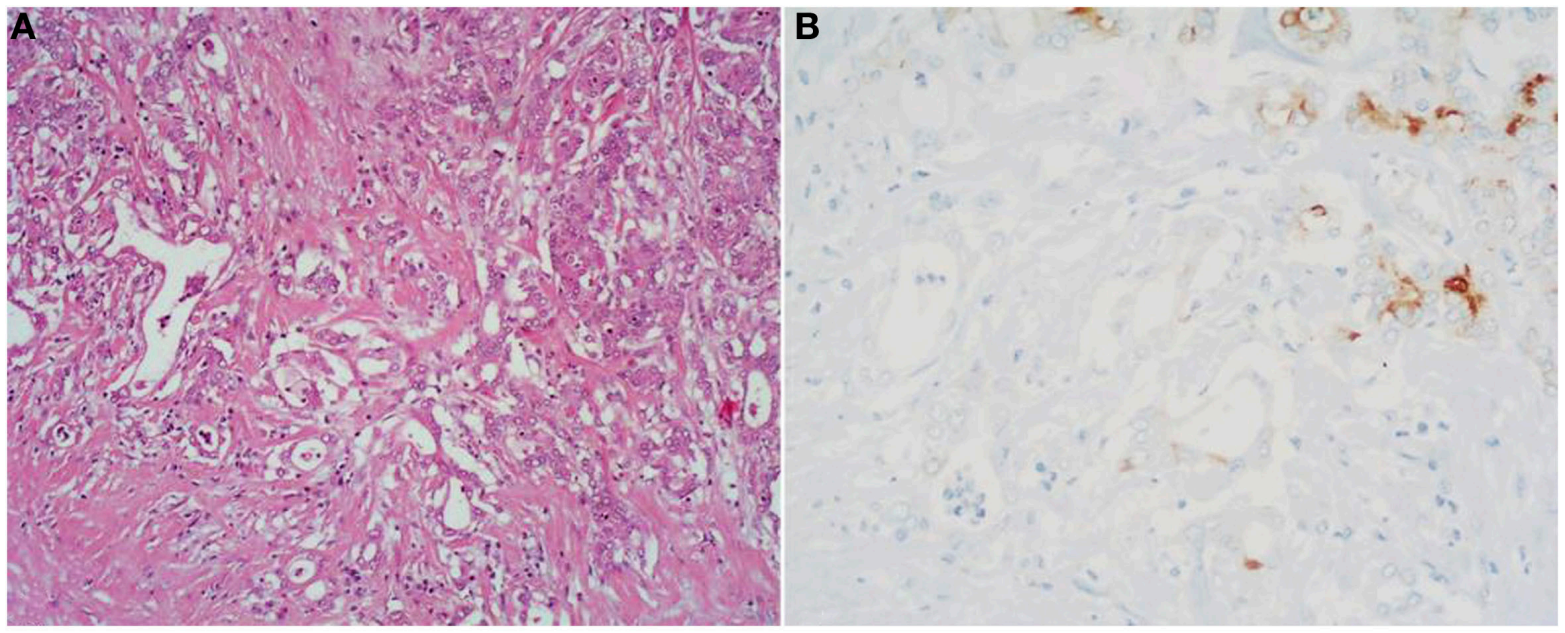
**(A)** Mixed acinar-ductal adenocarcinoma is a neoplasm showing both acinar (top right corner) and ductal differentiation. Neoplastic glands are generally surrounded by a desmoplastic stroma similarly to ordinary ductal adenocarcinomas. The acinar component is easily identifiable using acinar cell markers including BCL10 **(B)**.

These neoplasms show both acinar and ductal differentiation (Figure 6) with prevalence of the acinar component in most cases (27). Some display a conspicuous mucinous component in association with acinar cells. Others show neoplastic glands surrounded by desmoplastic stroma similar to those of ordinary ductal adenocarcinoma, combined with a more solid component showing acinar differentiation.

##### Mixed acinar-neuroendocrine-ductal carcinoma

This is a very rare and not well-documented variant of ACC with multiple directions of differentiation. Histologically, these neoplasms are very similar to mixed acinar-ductal carcinomas: the neuroendocrine component was not identifiable histologically in the reported cases, but was detected with immunostaining for chromogranin A and/or synaptophysin (27, 28).

### Ultrastructural features

**Figure 7:**
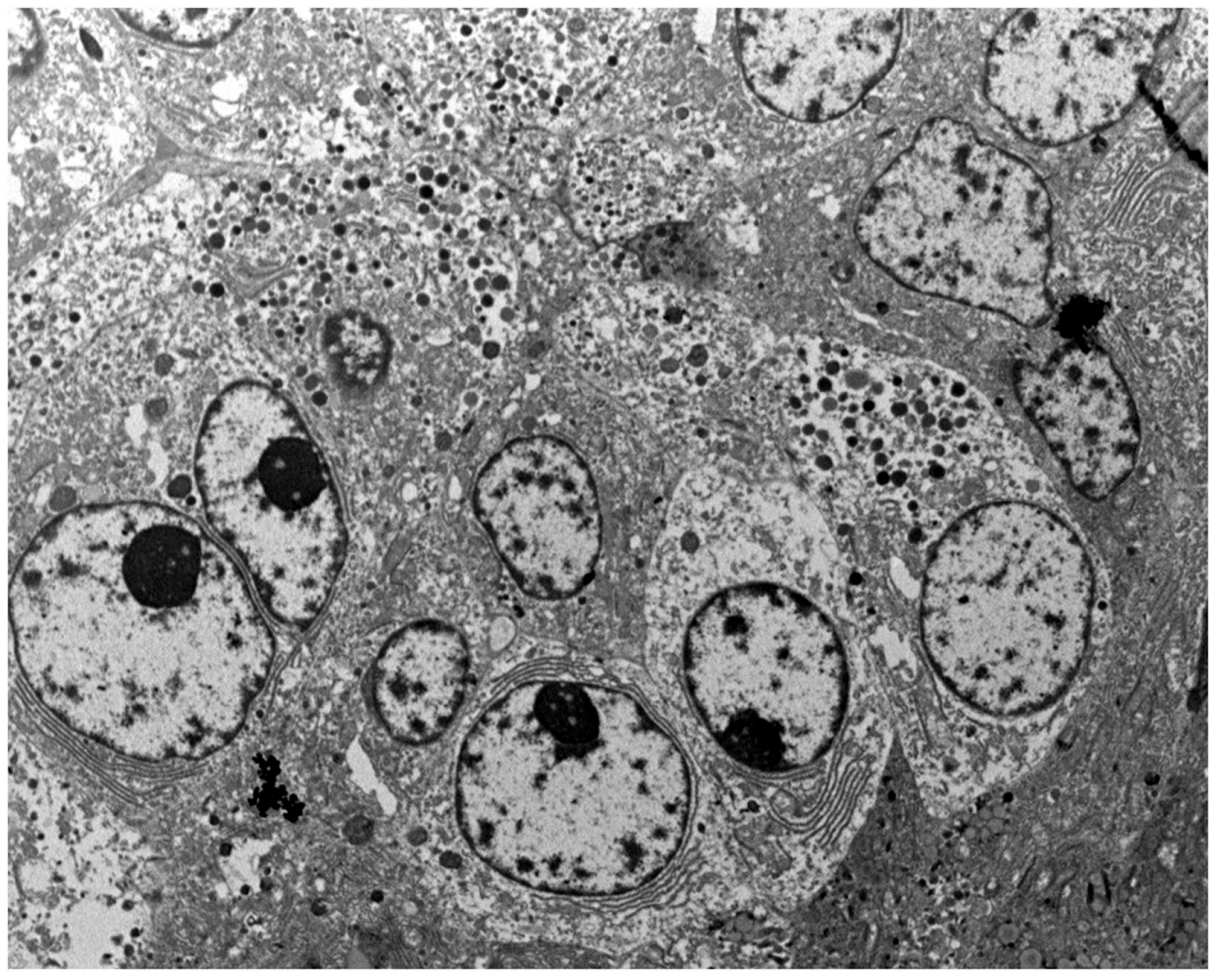
**At the ultrastructural level, tumor cells contain large electron-dense granules, abundant rough endoplasmic reticulum, well-developed Golgi complexes, and some mitochondria**.

Tumor cells often form acinar-like complexes. Tumor cells contain large electron-dense granules (Figure 7), abundant rough endoplasmic reticulum, well-developed Golgi complexes, and some mitochondria. Small microvilli are found in the apical surface. The majority of zymogen granules have a mean diameter of 400–500 nm and are homogeneous and round. The zymogen nature of these granules has been demonstrated with electron microscopy immunocytochemistry using anti-trypsin antibodies (29). A second granule type, the irregular fibrillary granule, resembling the zymogen granules of the fetal pancreas has also been detected (2).

### Immunohistochemical profile

Since the diagnostic hallmark of ACC is the demonstration of acinar differentiation, immunohistochemistry plays an indispensable diagnostic role in the demonstration of specific acinar cell products. Traditional antibodies used to identify acinar differentiation are those directed against trypsin, lipase, amylase, and CEL, but it is well known that they significantly differ in terms of sensitivity (4, 30, 31). It has recently been suggested that the monoclonal antibody directed against the COOH-terminal portion of the BCL10 protein (clone 331.3), which recognizes the COOH-terminal portion of CEL, is a useful tool for detecting ACCs, because it is highly specific and sensitive (31). The BCL10 immunoreactivity depends on the homology between the amino acid sequence 156 and 205 of the BCL10 protein (blast accession number: NP_003912.1) and the sequence between amino acid 564 and 608 of CEL (blast accession number: NP_001798.2) and does not depend on the true presence of the BCL10 protein in acinar cells (31). The utility in detecting acinar differentiation of the antibody directed against the COOH-terminal portion of BCL10 has been confirmed in further studies on both histological and cytological specimens (4, 32).

**Figure 8:**
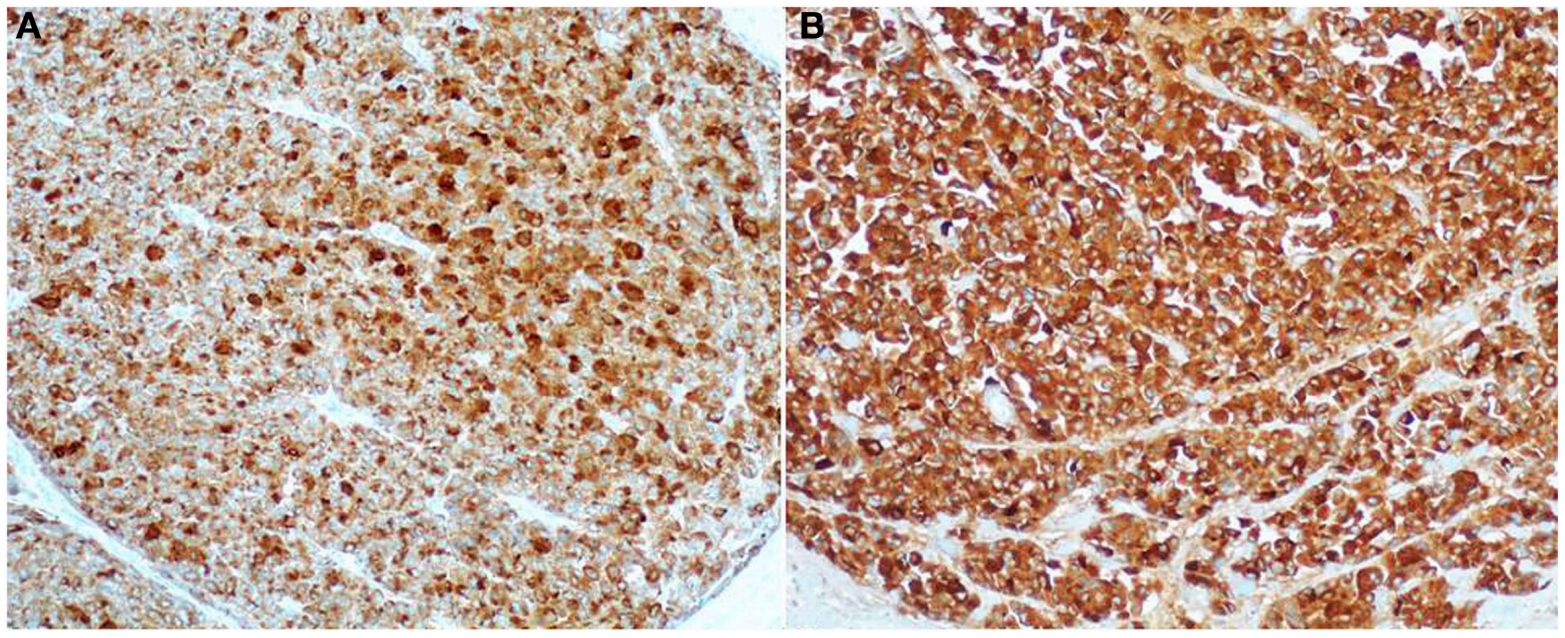
**Antibodies directed against trypsin (A) and BCL10 (B) are the best in terms of sensitivity in detecting acinar cell carcinoma**. The immunoreactivity is cytoplasmic and strong.

Among the various available antibodies, amylase is the least sensitive despite its wide expression in normal pancreatic acinar cells. Although the reason for this is not clear, amylase should not be considered as a useful diagnostic marker. In light of this observation, we suggest avoiding the use of the anti-amylase antibody in the diagnostic pathway of pancreatic ACCs, especially when only small bioptic specimens are available. Lipase expression, although more often found than amylase, is not detected in several cases. For this reason, we believe that lipase immunohistochemistry should only be included in the panel used to study surgical specimens for completeness. Our and other studies show that antibodies directed against trypsin and BCL10 (Figure 8) are the best in terms of sensitivity (2, 4, 30, 31). In our routine practice, we use both trypsin and BCL10 antibodies because it has been demonstrated that 15 and 4% of ACCs may be BCL10 and trypsin negative, respectively (4). In our hands, the simultaneous use of both antibodies allows the detection of approximately 100% of ACCs, therefore, avoiding the lack of recognition of those cases that only express one of these two markers.

**Figure 9:**
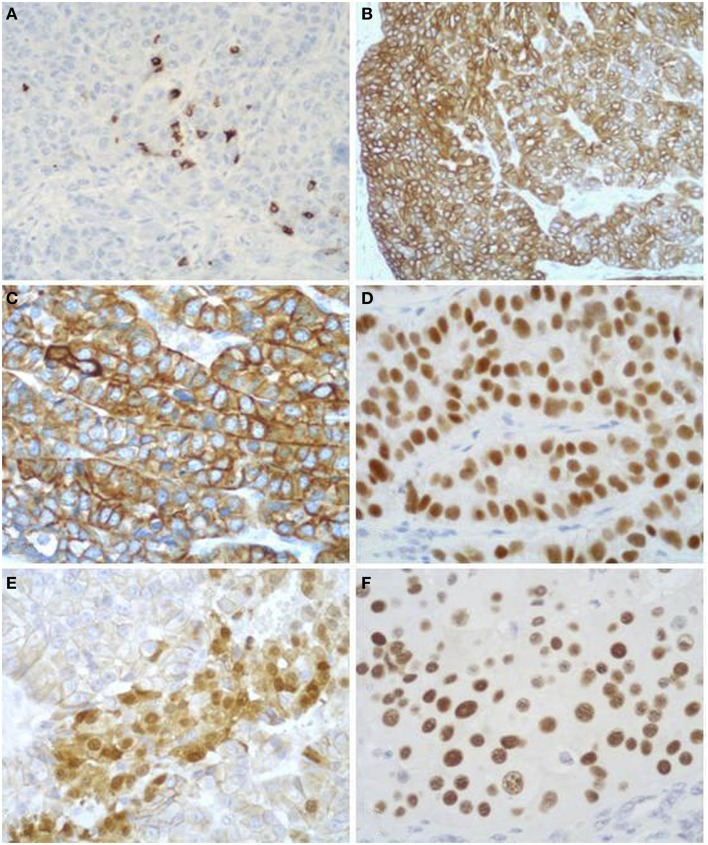
**Acinar cell carcinomas frequently show scattered chromogranin A-positive neuroendocrine cells (A), but for the diagnosis of MANEC they must represent at least 30% of the tumor tissue (see Figure 5)**. Acinar cell carcinomas may also express cytokeratin 7 **(B)** and cytokeratin 19 **(C)**, which are traditionally known markers of ductal adenocarcinomas. PDX1 immunoreactivity **(D)** is observed in about 90% of cases, while nuclear positivity for β-catenin **(E)** in about 15% of cases. Strong nuclear immunoreactivity for p53 **(F)** can be found in about 30% of carcinomas and its prognostic meaning is not clear, to date.

Scattered chromogranin A and/or synaptophysin positive neuroendocrine cells can be observed in several ACCs (Figure 9A). In about one-third of cases, they represent more than 30% of neoplastic cells and such ACCs are currently defined as MANECs following the criteria proposed in the 2010 WHO classification of tumors of the digestive system (24).

In addition to the acinar cell markers described above, ACCs may also express cytokeratin (CK) 7 and CK19 (Figures 9B,C) (4, 33, 34), traditionally known as markers of ductal adenocarcinomas. The knowledge that these two CKs can be expressed in at least a subset of ACCs should be taken into account during the diagnostic pathway for the differential diagnosis with ductal adenocarcinoma that should not be considered only on the basis of CK7 and CK19 immunoreactivity, independently of the morphological context.

Immunoreactivity for PDX1 (Figure 9D), a homeobox transcription factor involved in the regulation of pancreatic development, has recently been reported in ACCs (4, 35). This marker does not seem correlated with prognosis and it is not a marker of ACC when diagnosing a primary pancreatic neoplasm. It may be useful in the diagnostic pathway of metastatic lesions to document a possible pancreatic origin.

Nuclear expression of β-catenin (Figure 9E) can be found in about 10% of ACCs. This confirms that alterations in the β-catenin pathway are involved in a subset of ACCs (4, 36, 37). Its expression should also be considered for the differential diagnosis with SPTs (see the next paragraph).

Although the role of p53 in the tumorigenesis and progression of ACCs still remains to be elucidated and p53 nuclear immunoreactivity has not been found in some investigations (36, 38, 39), we have recently demonstrated that a subgroup of ACCs strongly express p53 (Figure 9F). Interestingly, patients with tumors showing more than 50% of p53-positive cells showed a trend of worse survival rate than those with tumors lacking or expressing p53 in <50% of neoplastic cells (4). However, further studies are needed to confirm the prognostic role of p53 expression.

### Differential diagnosis

The differential diagnosis between ACCs and other pancreatic tumor types should be based, first, on morphological features and, then, on immunohistochemical analyses, which are useful in demonstrating acinar differentiation.

The differential diagnosis with ductal adenocarcinoma is generally easy because of the different morphology of these two cancer types. However, in some particular cases of ACC with unusual morphology (spindle cell, clear cell, and pleomorphic cell variants), the use of immunohistochemistry is mandatory for the correct diagnosis. ACCs express acinar markers (trypsin and BCL10 are the most sensitive and specific), which are lacking in ductal adenocarcinoma. It is worth noting that ACCs may express CK7 and CK19, which are not consequently specific indicators of a ductal origin. For this reason, the value of CK7 and CK19 expression in a pancreatic tumor should be evaluated with caution.

The differential diagnosis with PanNETs may be more difficult because of some morphological similarities between ACCs and PanNETs. However, the presence of a high-mitotic index, abundant necrosis, clearly evident nucleoli in apparently well to moderately differentiated cells with abundant eosinophilic cytoplasm, should give rise to the suspicion of an ACC. Immunohistochemistry is useful, but it must be used with caution. The use of general neuroendocrine markers including chromogranin A and synaptophysin alone may be risky because several ACCs show at least scattered neuroendocrine cells, which, in 30% of cases, are particularly abundant (MANECs). For this reason, if an ACC is suspected, the demonstration of acinar cell products in mandatory.

The differential diagnosis with SPT may be difficult, although this tumor is more frequent in females and rarely shows abundant necrosis. SPTs typically show nuclear immunoreactivity for β-catenin and strong expression of CD10 that, however, can also be found in about 10 and 60% of ACCs, respectively (4, 33). For this reason, these two markers should not be used alone but in association with acinar-specific ones such as trypsin and BCL10.

Pancreatoblastoma (PB) is a malignant epithelial neoplasm showing multiple lines of differentiation including acinar differentiation. Consequently, the differential diagnosis with ACC may be difficult. The presence of squamoid nests in PB is the histological hallmark that distinguishes between the two entities.

Acinar cell cystadenocarcinoma needs to be distinguished from acinar cell cystadenoma, which is a rare benign cystic neoplasm lined by cells showing acinar differentiation. In acinar cell cystadenocarcinoma, the epithelium lining the cysts is more complex and tumor cells are less well polarized, atypical with a well evident single nucleolus. Moreover, areas of solid growth with necrosis and infiltration into the stroma support the diagnosis of malignancy (40).

In addition to the differential diagnosis with other primary pancreatic neoplasms, liver metastases from pancreatic ACCs need to be distinguished from primary liver ACCs (41, 42). Since the morphological features are identical between the two entities, an accurate radiological investigation and intra-operative examination are mandatory. It is worth noting that primary liver ACCs are generally solitary neoplasms, while metastases from pancreatic ACCs are generally multiple and associated with metastatic spread outside the liver in most of cases (4, 42). This clinical context should be taken into account when diagnosing a suspected primary liver ACC.

### Molecular pathology

The molecular mechanisms involved in the onset and progression of ACCs are poorly known, although in the last 10 years, several attempts have been made, using different technical methods, to better understand the molecular biology of such cancers. The main difficulty in studying the molecular profile of ACCs is their rarity and, consequently, the difficulty in collecting a significantly large number of cases. In general, the most common molecular alterations of ductal adenocarcinomas including mutations of *KRAS*, *TP53*, *DPC4*, and *p16* genes are absent or rare in ACCs, while losses of chromosome 11p, alterations in the APC/β-catenin pathway, and loss of DCC expression have been observed in several cases. In the present section, we will review the molecular alterations investigated in adult ACCs (Table 1).

**Table 1:** **Summary of gene alterations in pancreatic acinar cell carcinomas and mixed acinar-neuroendocrine carcinomas described in the medical literature**.

Gene	Mutation	Reference
*KRAS*	2/196 (1%)	(37, 38, 43–49)
*EGFR*	0/57	(48)
*BRAF*	1/17 (5.9%)	(47)
*p16*	0/6	(43)
*BRCA2*	4/30	(47, 50)
*DPC4/SMAD4*	11/59 (18.6%)	(47, 49)
*TP53*	12/133 (12%)	(43, 44, 46, 47, 49, 51, 52)
*APC*	8/99 (8%)	(36, 37, 47, 49)
*CTNNB1*	5/70 (7%)	(36, 37, 49)
**Gene**	**Deletion**	**Reference**
*TP53*	46/187 (25%)	(39, 43, 45, 47–49, 52, 53)
*BRCA2*	1/3 (33%)	(53)
*APC*	12/25 (48%)	(37)
**Gene**	**Methylation**	**Reference**
*APC*	24/43 (56%)	(37)
*RASSF1*	26/43 (60%)	(37)

#### KRAS Mutations

While *KRAS* mutations are commonly found in ductal adenocarcinomas (43), they are exceedingly rare in ACCs. *KRAS* mutations have been investigated in 9 studies including 196 ACCs/MANECs and they were only found in 2 cases, representing 1% (37, 38, 43–49).

#### EGFR Mutations

Mutational analysis of *EGFR* (exons 18–21) has been investigated recently in 57 ACCs and no mutations have been found (48). In the same study, EGFR immunoreactivity at the cell membrane level was observed in 19/45 (42%) cases. The *EGFR* status may be interesting for the possible use of anti-EGFR-directed therapy. However, there is currently not enough data to suggest the possible response to this target therapy.

#### BRAF Alterations

*BRAF* mutations have been investigated in only two studies using either whole-exome sequencing analysis (47) or immunohistochemistry with the anti-BRAF V600E antibody (48). *BRAF* mutations were found in two mixed acinar-ductal carcinomas and in only 1 out of 17 pure ACCs (47). Among the 42 immunohistochemically investigated ACCs none were positive for the anti-BRAF antibody (48). Although *BRAF* mutations are very rare, it has recently been demonstrated that about 23% of ACCs harbor rearrangements involving *BRAF* and *RAF1* and the most prevalent fusion was *SND1-BRAF*. Interestingly, *SND1-BRAF* transformed cells were sensitive to treatment with MEK inhibitors (trametinib), suggesting a new therapeutic approach to ACCs (49).

#### p16 Mutation

*p16* mutation does not seem to be involved in ACCs, although only a few cases have been investigated. Moore and coworkers did not find any *p16* mutations in 6 ACCs (43). Although these data suggest that p16 is not involved in ACCs tumorigenesis, further studies enrolling many more cases are needed to finally clarify the role of this gene.

#### DPC4/SMAD4 Alterations

Although traditionally *DPC4/SMAD4* mutations have been believed to be absent in ACCs, recent investigations have demonstrated mutations or truncations of these genes in at least a subset of cases using different methodological approaches including mutational analysis (43), whole-exome sequencing analysis (47), NGS-based genomic profiling (49), and immunohistochemistry (36, 39, 43, 46). *DPC4/SMAD4* mutations were found in 11/59 (18.6%) ACCs/MANECs (47, 49), while lack of DPC4 immunoreactivity was observed in 1 out of 35 (2.8%) cases investigated (36, 39, 43, 46).

#### TP53 Mutations and p53 Immunohistochemical Expression

p53 expression in ACCs was immunohistochemically investigated for the first time in 1993 by Hoorens and coworkers using a polyclonal antibody, which did not identify any nuclear immunoreactivity (38). Since then three other studies, using the monoclonal DO-7 or DO-1 antibodies, have investigated the immunohistochemical nuclear expression of p53, which was found in 10/85 (11.7%) cases (4, 36, 39). However, the comparison of such investigations is quite difficult because of the different antibodies employed, different immunohistochemical methods, and different cut-offs used to define a tumor as positive. We have recently used a very high cut-off (50% of positive cells) to consider a tumor as positive. This choice was derived from the fact that it has been demonstrated that high cut-off of p53 protein expression accurately identifies gene mutation, thus consistently reducing false-positive immunoreactivity (4). Because the first published papers, mainly based on immunohistochemical findings, did not demonstrate p53 alterations, it has traditionally been considered that, unlike pancreatic ductal adenocarcinomas, ACCs do not harbor *TP53* mutations. However, after accurately analyzing the seven studies in which *TP53* mutations were investigated, it clearly appears that it is not as rare as believed initially. Indeed, *TP53* mutations have been identified in 12 out of 133 (12%) ACCs (43, 44, 46, 47, 49, 51, 52). In addition to mutations, *TP53* gene loss has also been documented in different studies (43, 49, 52, 53). Interestingly, p53 immunoreactivity showed a trend of poor survival (4) and the concomitant presence of both *TP53* mutations and gene loss correlated with a worse prognosis (52). However, further studies are needed to clarify the potential role of p53 in the progression and aggressiveness of ACCs.

#### BRCA2 Alterations

*BRCA2* mutations were found in 4/30 ACCs (47, 50). In the study by Furukawa et al., the reported *BRCA2* mutations were either somatic or germline premature termination mutations associated with loss of the normal wild-type allele and lack of BRCA2 immunoreactivity. Interestingly, one of these patients presented complete remission of liver metastases after cisplatinum-based chemotherapy (50). Alterations of *BRCA2* including truncation and loss were identified in 6/29 and 1/3 ACCs, respectively (49, 53). In one study, *BRCA2* amplification has been found in one out of five ACCs (46).

#### APC/β-Catenin Pathway Alterations

Alterations in the APC/β-catenin pathway have been well-documented and include mutations of both *APC* and *CTNNB1* genes. *APC* mutations have been investigated in 4 studies including 99 ACC/MANECs. They were found in eight cases (8%). *CTNNB1* mutations have been demonstrated in 5/70 (7%) ACC/MANECs investigated in three different studies (36, 37, 49), while abnormal nuclear β-catenin immunoreactivity has been documented in 5/39 (12.8%) cases investigated in two studies (4, 46). However, it has recently been demonstrated that the most frequent alterations of the *APC* gene are mainly due to gene loss and/or promoter hypermethylation rather than *APC* mutations. *APC* loss and hypermethylation were found in 48 and 56% of ACC/MANECs, respectively, whereas *APC* mutations were detected in only 7% (37). Interestingly, loss and methylation of *APC* gene were also found in normal appearing non-neoplastic pancreatic tissues adjacent to ACCs, suggesting that the “field cancerization” phenomenon, which has been described in prostate, head and neck, colon, esophageal, lung, and breast carcinomas (54) may also exist for pancreatic ACCs (37). Another interesting aspect of *APC* alterations in ACCs is the well-known correlation between chromosome instability and loss of *APC* function, which, being involved in mitosis regulation, leads to polyploidy. Interestingly, low APC mRNA levels were directly correlated to high frequency of copy number alterations and chromosome instability (37, 48).

In addition to these direct evidences, which demonstrate the role of *APC* gene in the pathogenesis of pancreatic ACCs, there are additional indirect experimental findings that suggest that this gene is involved in the pathogenesis of such carcinomas and, in general, in the biology of normal pancreatic acinar cells. *APC* gene inactivation, in pancreatic epithelial cells of mouse embryos, induced pancreatomegaly due to selective proliferation of acinar cells between birth and 6 months of age (55). Moreover, mice constitutively null for functional p53 and heterozygous for *APC* mutations developed dysplastic acinar foci and ACCs (56). Whereas in DNA methyltransferase (Dnmt) 1 hypomorphic mice, reduced methylation of tumor suppressor genes, mainly *APC*, leads to a reduction of pancreatic ACC development (57).

#### Chromosomes Abnormalities

The first description of chromosome abnormalities in ACCs was reported in 2002 by Abraham and coworkers who described the allelic loss of chromosome 11p in 50% of the cases they investigated (36). Since then, chromosome gains and losses have been investigated using different molecular approaches including comparative genomic hybridization (CGH), fluorescence *in situ* hybridization (FISH), multiple ligation probe amplification (MLPA), loss of heterozygosis (LOH) analysis using PCR, and whole-exome sequencing analysis (37, 39, 47, 48, 53). From the global analysis of the findings reported in the literature, it appears that pancreatic ACCs show chromosome instability characterized by high degrees of allelic loss and gains. The more frequently involved regions as losses included 1p, 3p, 5q, 6q, 8p, 9p, 11, 17p, and 18q, while the gained regions mainly were 1q, 7, 8q, 12, 17q, and 20q (37, 45, 47, 48). Interestingly, a hierarchical clustering of CGH findings did not find differences between pure ACCs, cystic ACCs, and MANECs indicating that these subtypes have the same cytogenetic background. Moreover, although some cytogenetic similarities between ACCs and ductal adenocarcinomas have been observed, the cytogenetic profile of ACCs is globally different from that of ductal adenocarcinomas confirming that these two types of exocrine pancreatic cancers are two different entities also in terms of cytogenetic alterations (48). Several chromosomal imbalances have also been associated with tumor size and metastatic spread suggesting a possible role of chromosomal abnormalities in the progression and aggressiveness of ACCs. A very recent and interesting finding regards the amplification of c-MYC, located at the 8q24 locus, which was found in 17% of ACCs, even if its role in biological aggressiveness remains to be clarified (48). Loss of 18q was one of the most frequent chromosome abnormalities found in a recent study on ACC (48). This loss was correlated with the loss or significant reduction of deleted in colorectal carcinoma (DCC) protein and has been considered as an early step in the development of ACCs.

#### Methylation Profile

Aberrant methylation of CpG islands is a well-known mechanism associated with the silencing of cancer-related genes in several cancers including pancreatic adenocarcinomas (58, 59) and PanNETs (60). In the literature, there is only one study, which, analyzing the promoter methylation status of 34 tumor suppressor genes, evaluated the methylation profile of ACCs (37). In general, ACCs showed low levels of hypermethylation and no tumors characterized by concerted hypermethylation at multiple loci were identified among 55 ACC/MANECs investigated. Consequently, the so-called “CpG island methylator phenotype (CIMP)” described not only in colorectal cancers but also in pancreatic ductal adenocarcinomas and PanNETs (60, 61) seems not to be present in pancreatic ACCs. However, some genes, including *RASSF1* and *APC*, frequently were found to be methylated. Interestingly, *RASSF1* promoter methylation was found also in non-neoplastic pancreatic tissues adjacent to ACCs (37).

#### Microsatellite Instability

Acinar cell carcinoma is an unusual tumor found in the context of Lynch syndrome with only four cases described in the literature (12, 13, 62). However, microsatellite instability has been evaluated in five different studies since Abraham and coworkers described three unstable ACCs in 2002 (13, 36, 46, 48, 62). Microsatellite instability has been investigated in a total of 159 ACCs and 13 of them (8.2%) were found to be unstable. The relationship between MSI and MLH1 methylation has never been investigated in pancreatic ACCs. Unstable ACCs did not show peculiar morphological features useful for their identification (13). It is also worth noting that there are not enough clinicopathological data demonstrating a different survival of unstable ACCs, compared to stable ones. Furthermore, there are no clinical trials suggesting a different therapeutic approach to this subset of ACCs to date.

### Prognostic factors

The prognostic meaning of several morphological features, immunohistochemical markers, and molecular abnormalities has been investigated in recent years. Some markers, including vascular invasion, perineural infiltration, CK19 immunoreactivity, and p53 expression in more than 50% of neoplastic cells, showed a trend of worse survival but not reaching statistical significance (4). Other markers such as tumor size, lymph node and distant metastases, *RASSF1* methylation, and *STK11* and *TP53* copy number alterations were statistically associated with prognosis in univariate analysis (4, 37). However, in multivariate analysis, only UICC stage proved to be an independent prognostic factor. Interestingly, the best prognostic stratification of patients was observed by grouping together stage I with stage II, and stage III with stage IV, suggesting a simplification of UICC staging for pancreatic ACCs (4).

## Acinar Cell Carcinoma in Infancy

### Epidemiology

Exocrine pancreatic tumors including ductal adenocarcinomas, ACCs, SPTs, and PBs are rare in children and adolescents. The rarity of such tumors has been the cause of the small number of cases analyzed in collective studies (63–67). The study with the largest number of patients examined the SEER data and contained significant demographic and statistical analyses (63). The incidence rate of pancreatic tumors in 0- to 19-year-old subjects was 0.018/100,000 inhabitants per year (adjusted to the 2000 US standard population) in the SEER database, 0.20 in the TREP report and 0.018 in a German study (54, 63, 67). The incidence rates, age adjusted to the 2000 US standard population for tumor histology, were 0.003 for ductal adenocarcinomas, 0.003 for ACCs, 0.004 for PBs, 0.005 for SPTs, and 0.007 for PanNETs (63). From the data reported in three studies, SPTs represented the most frequent pancreatic neoplasm in pediatric population accounting for 29.8% of the cases, followed by PanNETs (24.7%), PBs (23.7%), ACCs (7.2%), ductal adenocarcinomas (7.2%), and non-epithelial neoplasms (7.2%) (63–65). From the same studies, it appeared that 86.2% of SPTs occurred in females whereas males had a higher incidence of ductal adenocarcinomas (71.4%). Of the 23 PBs, 16 (69.6%) were diagnosed in patients younger than 10 years, while 96.5% of SPTs, 71.4% of ductal adenocarcinomas, and 54.1% of PanNETs occurred in children 10 years or older. Among the 29 cases of pediatric ACCs reported in 20 papers from 1970 to 2014, there were 18 males and 11 females with an average age of 9.57 years (range 3–16 years); 14 carcinomas occurred in children 10 years or older.

### Localization

Pediatric ACCs may involve any portion of the pancreas but are more common in the tail (41% of cases), followed by the head (32%), the body (13.5), the body-tail (9%), and the head-body (4.5%).

### Clinical features

Most cases present with vague abdominal pain or discomfort and abdominal swelling or mass. Jaundice is not a part of the usual clinical presentation. Some children may have nausea and vomiting or mild fever and anemia. It must be underlined that, unlike in adult cases, none of the ACC reported in children presented with polyarthralgia, panniculitis, or subcutaneous fat necrosis due to lipase release by the tumor into the circulation (2). Serum AFP levels were elevated in all pediatric cases of ACCs in which it was measured (10, 67) and it has been hypothesized that AFP production in pancreatic neoplasms is related to acinar differentiation (10). In 3 of 29 pediatric ACCs, the most significant clinical manifestation was Cushing’s syndrome due to ectopic production of ACTH by tumor cells (11, 68, 69).

### Macroscopy

Macroscopically, pediatric ACCs are usually large at the time of presentation, with an average diameter of 11.8 cm (range 3.2–20 cm). They are well circumscribed, fleshy, pink to tan, and generally contain areas of necrosis and/or hemorrhage. Cystic variants have been described less frequently (70).

### Histological and immunohistochemical features

**Figure 10:**
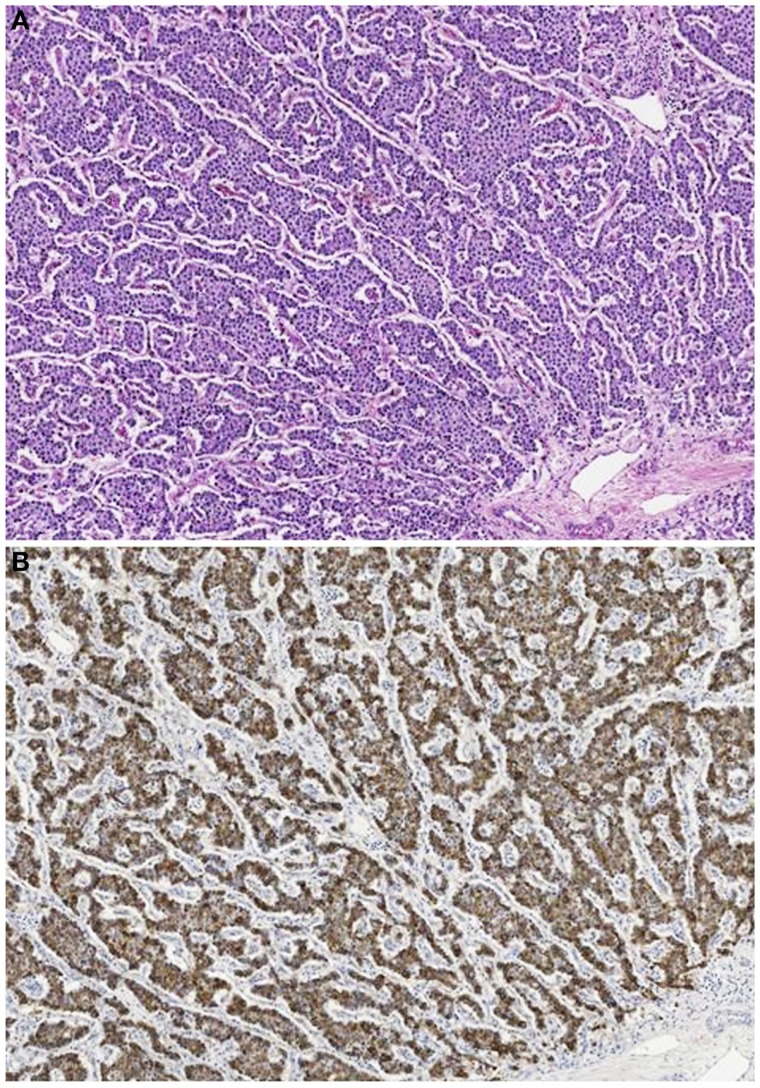
**Acinar cell carcinoma in a 1-year-old boy characterized by a trabecular growth (A) resembling that of a pancreatic neuroendocrine tumor**. However, tumor cells are strongly immunoreactivity for trypsin **(B)**.

Acinar cell carcinomas in children show the same histological features and immunohistochemical reactivity of those of adults (Figure 10). Among the 29 cases reported in the literature, 22 were pure ACCs, 4 MANECs, 1 acinar cell cystoadenocarcinoma, 1 mixed acinar-ductal carcinoma, and 1 mixed acinar-neuroendocrine-ductal adenocarcinoma. The cases of ACCs associated with Cushing’s syndrome were characterized by a surprisingly scant neuroendocrine component weakly immunoreactive for ACTH and general neuroendocrine markers including chromogranin A and synaptophysin (11, 68, 69).

### Differential diagnosis

Acinar cell carcinoma in the pediatric population may closely resemble a PanNET and has overlapping features with PB and SPT. Squamoid nests, absent in ACC, are considered the elements required for the diagnosis of PB (71). Unlike ACCs that show only focal or zonal expression of neuroendocrine markers, PanNETs show diffuse expression of these markers and lack the expression of trypsin, chymotrypsin, lipase, and BCL10. In cases of SPTs lacking the characteristic pseudopapillary structures and mimicking ACCs, the immunohistochemical expression of CD10 and vimentin, the nuclear labeling of β-catenin, and the negativity for exocrine enzymes favor the diagnosis of SPT.

### Molecular pathology

The only study examining molecular alterations in ACCs in children is by Abraham and coworkers (36): in two pediatric cases, no allelic loss of chromosome 11p and no mutations in the APC/β-catenin pathway were detected, unlike some of the adult cases. High frequencies of both allelic loss of ­chromosome 11p and mutations in the APC/β-catenin pathway were detected in PBs.

### Prognosis and predictive factors

Metastases have been reported in 41% of the 29 pediatric ACCs described in the literature, being loco-regional in 26% of the cases and distant in 22%. Patients underwent only surgical resection in 43% of cases, surgical resection combined with chemotherapy and/or radiotherapy in 36% of cases and palliative chemotherapy in 21% of cases. After chemotherapy, 45% of the patients were alive with no evidence of disease with an average follow-up time of 25.5 months (from 3 to 132 months), 13.7% were alive with disease with an average follow-up time of 14.7 months, 27.5% died of disease with an average survival of 34.4 months. The above reported data confirm the suggestion that pediatric patients with ACCs may have a better prognosis than adults (2).

In conclusion, ACC is a very rare pancreatic neoplasm in children that must be distinguished from PanNET, PB, and SPT. The clinical evolution of this neoplasm in children seems to be better than that observed in adults. Pediatric pathologists should consider this neoplasm in the differential diagnosis of primary pancreatic masses in children.

## Author Contributions

All the three authors designed and participated in writing the present review.

## Conflict of Interest Statement

The authors declare that the research was conducted in the absence of any commercial or financial relationships that could be construed as a potential conflict of interest.
